# Sneaky copulations by subordinate males suggest direct fitness benefits from male–male associations in spotted bowerbirds (*Ptilonorhynchus maculatus*)

**DOI:** 10.1111/eth.13336

**Published:** 2022-10-09

**Authors:** Giovanni Spezie, Leonida Fusani

**Affiliations:** ^1^ Konrad Lorenz Institute of Ethology University of Veterinary Medicine Vienna Austria; ^2^ Department of Behavioural and Cognitive Biology University of Vienna Vienna Austria

**Keywords:** alternative reproductive tactic, bowerbird, cheating, courtship coalition, direct fitness benefits, sneaky copulation

## Abstract

Male spotted bowerbirds (*Ptilonorhynchus maculatus*) build and defend a structure of sticks and straw—the bower—decorated with colourful objects to attract mates during the breeding season. Specific non‐territorial, subordinate males are tolerated by resident males at bowers over multiple breeding seasons. Prior research showed that these male–male associations exhibit attributes of coalitionary behaviour and that subordinate males gain delayed benefits from associating with bower owners, namely future bower inheritance. Yet, it remained unclear whether subordinate males may additionally gain direct fitness benefits from attending established bowers. Here, we report on four separate instances of sneaky copulations (or attempts of copulating) by subordinate males at resident males' bowers. Multiple non‐resident males disrupted the ongoing copulations between the bower owner and a receptive female, and these events were followed by violent aggressive interactions. These observations shed new light on same‐sex social dynamics in spotted bowerbirds and support the hypothesis that subordinate males are sexually mature individuals that occasionally obtain access to females while attending established bowers. We discuss these findings in light of the literature on male courtship coalitions and agonistic behaviour in bowerbirds, and highlight further aspects of subordinate behaviour that require empirical investigation.

## Graphical abstract

Resident male spotted bowerbirds tolerate the presence of non‐territorial, subordinate males at their display arenas. We report in this paper on four separate instances of sneaky copulations by subordinate males at adults' bowers. These novel observations suggest that subordinate males gain direct fitness benefits by associating with older bower owners at established bowers. The implications of this finding for male‐male associations and coalitionary behaviour in this species are discussed in detail.

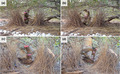

## 1 INTRODUCTION

Animals with non‐resource‐based polygynous (NRP) mating systems engage in conspicuous courtship displays on individual or collective arenas (Höglund & Alatalo, 1995; Johnsgard, 1994). A number of species exhibit various degrees of male–male associations on display arenas, ranging from facultative to obligate forms of cooperative courtship. These coalitions have been described across taxa, particularly in NRP birds [manakins (DuVal, 2007a; Foster, 1977), grouse (Wiley, 1991), peacocks (Petrie et al., 1999), wild turkeys (Krakauer, 2005), bowerbirds (Madden, 2008)]. A common feature of most courtship coalitions in birds is that a dominant “alpha” male obtains all or most copulations, while subordinate “beta” males forgo breeding and gain no—or very limited—access to mates (DuVal, 2007a, 2007b; Foster, 1977; McDonald, 1989). Sacrificing reproductive potential to associate with other males may appear paradoxical, and this evolutionary conundrum has been discussed in a number of prior studies (Díaz‐Muñoz et al., 2014; Nonacs & Hager, 2011; Prum, 1994; Wiley & Rabenold, 1984). In particular, empirical and theoretical research has sought to elucidate the benefits that may accrue to subordinate males from establishing multi‐male partnerships (DuVal, 2013; Kokko & Johnstone, 1999; Olson & Blumstein, 2009).

Prior research showed that subordinate males in some species gain *indirect* fitness benefits by associating with closely related individuals (Hamilton, 1964). For instance, wild turkeys (*Meleagris gallopavo*) establish partnerships with kin on display arenas that increase their ability to monopolize access to females (Krakauer, 2005; Krakauer & DuVal, 2012). Second, subordinate males may obtain *delayed* direct benefits via an increase in their probability of gaining future alpha position, for example by increasing the chances of future arena inheritance or survival (Kokko & Johnstone, 1999; McDonald & Potts, 1994) or via the acquisition of skills required for successful sexual signalling (Díaz‐Muñoz et al., 2014; DuVal, 2013; Selander, 1965; Skutch, 1961). Finally, secondary males may gain *immediate* direct benefits via opportunistic and/or coercive (hereafter “sneaky”) copulations (Ortega & Arita, 2002). Though selection is expected to greatly favour non‐dominant males that pursue such alternative reproductive strategies (Gross, 1996; Taborsky et al., 2008), sneaky copulations appear to be uncommon in NRP species with facultative or obligate male–male coalitions (Boyle & Shogren, 2019; Rivers & DuVal, 2020). Sneaky copulations may be rare and inconspicuous because of the costs associated with retaliation and, therefore, difficult to document. In alternative, subordinate males may truly forego reproduction because of the strict control exerted by dominant males on their access to mates, or because inexperienced males are unattractive to females (Trainer et al., 2002) or physiologically unable to achieve successful fertilizations.

In a number of species, it is not known whether subordinate males are indeed sexually mature. Most NRP birds retain immature secondary sexual characteristics for several years after hatching (Foster, 1987; Hawkins et al., 2012; Schaedler et al., 2021). For instance, long‐tail manakins attain definitive plumage in their fifth year—the longest delay yet documented in a manakin species (Doucet et al., 2007). Satin bowerbirds (*Ptilonorhynchus violaceus*) moult into adult plumage in their seventh year after intermediate plumage stages (Vellenga, 1980). Although systematic information on subordinate reproductive physiology is rarely available, plumage development and maturation of reproductive organs do not seem to coincide. Mature gonads and viable sperm were found in males with pre‐definitive plumage in at least three NRP species [satin bowerbirds (*Ptilonorhynchus violaceus*) (Marshall, 1954), white‐ruffed manakins (*Corapipo altera*) (Aldrich & Bole, 1937) and in dusky grouse (*Dendragapus obscrus*) (Hannon et al., 1979)]. Thus, in spite of the apparent potential to carry out successful fertilizations, for a variety of NRP species it remains unclear whether subordinate birds gain direct fitness benefits from attending established arenas.

Bowerbirds (Ptilonorhynchidae) are NRP birds found in Australia and New Guinea (Frith & Frith, 2004). Male bowerbirds build and defend a structure of sticks and straw—the bower—which is decorated with species‐specific objects to attract mates during the breeding season. Some forms of male–male associations between resident bower owners and non‐territorial visitors have been described in at least three species in this family [golden bowerbirds *Prionodura newtoniana* (Frith & Frith, 2000a, 2000b); satin bowerbirds *Ptilonorhynchus violaceus* (Maxwell, 1999; Vellenga, 1970, 1986); spotted bowerbirds *Ptilonorhynchus maculatus* (Isden, 2014; Spezie & Fusani, 2022); reviewed in Madden (2008)]. In spotted bowerbirds, these non‐territorial (hereafter “subordinate”) males are tolerated at established bowers over multiple breeding seasons, and engage in bower building and displaying, even in the presence of the bower owner (Spezie & Fusani, 2022). Prior research in this species showed that these male–male associations exhibit attributes of coalitionary behaviour, are stable across breeding seasons and that subordinate males are more likely to inherit their partner's display arena (Isden, 2014; Spezie & Fusani, 2022). In bowerbirds, it remained unclear whether subordinate males may additionally gain immediate direct benefits—that is a share of copulations—from attending established bowers. Here, we present four separate observations of sneaky copulations (or attempts of copulating) by non‐resident males at established bowers in spotted bowerbirds (*Ptilonorhynchus maculatus*) during one breeding season in 2018. We discuss the implications of our findings for male–male social dynamics in bowerbirds, also in light of the current knowledge on disruptive and cheating behaviour in bowerbirds and other NRP systems with cooperative courtship.

## 2 METHODS

We conducted field activities at Taunton National Park (23.54989° S; 149.24088° E) between July and December in 2018 and 2019. Birds were caught using mist‐nets and marked with individual combinations of colour bands. As subordinate males are not distinguishable from females via morphological features (Madden et al., 2004), blood samples were drawn upon capture for genetic sexing. Spotted bowerbirds can only be assigned to adult (2+) or juvenile (first year) age categories based on morphology (Higgins et al., 2006). Among the subordinate males considered in the present study, only one individual was identified as being younger than two years based on plumage and morphology (Higgins et al., 2006), and none of the banded subordinate males that attempted sneaky copulations had juvenile morphological features.

We set up motion‐activated camera traps at 14 bowers and video‐recorded courtship behaviour and social interactions among males. In both breeding seasons, all resident males except one individual had one or more “regular” subordinate males (i.e. observed repeatedly at a bower throughout the breeding season) attending their bower for an average 28.4 ± 13.5% (standard deviation, SD; *N* = 14 bowers) of total recording time, in line with previous reports (Isden, 2014; Madden, 2008). Our observations include attempted copulations by unbanded birds, and these birds were characterized as “presumably males” based on behavioural cues. Among the individuals for which genetic data were available, all the individuals that exhibited male‐specific behaviours turned out to be males after genetic sexing; therefore, it is very likely that all other non‐tested individuals that exhibited male‐specific behaviours were males as well. Moreover, based on the reactions of the bower owners (see below), it seems most likely that the attempted copulations were from males. Finally, we never observed female–female copulations in our population of spotted bowerbirds, thus it seems very unlikely that unbanded birds attempting or disrupting copulations were females.

In 2018, rainfall was below long‐term averages, and in 2019, our study site experienced a major drought (Bureau of Meteorology, Australian Government), with most bowerbirds gradually decreasing activity at bowers and finally abandoning display arenas later in the breeding season (October–December 2019). Thus, we did not observe any copulations by either bower owners or subordinate males in 2019. All values reported below are means ± standard deviation [SD].

## 3 RESULTS

Our observations resulted from the continuous video‐recording at 14 bowers using motion‐activated cameras, for a total duration of approximately 24,500 h in 2018 and 2019 [total duration of time recorded: 2018 = 10,815 h (mean per bower = 676 ± 146 h); 2019 = 13,634 h (mean per bower = 852 ± 324 h)]. If we only consider the 2018 video‐recording duration—given that no copulations took place in 2019—the rate of sneaky copulations in our study population is <0.0004 per hour of video‐recording (or <0.0006 per hour of video‐recording, if we include the two additional observations). For comparison, we observed *N* = 67 copulations by bower owners in 2018, with a copulation rate of 0.006 copulations per hour of video‐recording.

### 3.1 Observation of sneaky copulations by subordinate males in the presence of the bower owner

During the 2018 breeding season, we observed three separate instances of sneaky copulations by non‐resident males during female visits in the presence of the bower owner. On 10 November 2018 at 05:51 (Observation 1) at a bower identified as #50 (23.59349° S; 149.24815° E), we video‐recorded an unbanded bird (presumably female) landing on the display arena and positioning itself in the crouching position typical of copulation solicitation in female bowerbirds (Patricelli et al., 2002; Video S1). A “regular” subordinate male identified as BNY‐RPM rapidly flew in the bower and attempted a copulation, but was soon after knocked off the female by the bower owner. A second attempt of copulating with the female by BNY‐RPM was disrupted by a fourth unbanded bird, which landed on the bower and started a chaotic clash with BNY‐RPM; both birds attempted to mount and copulate with the female. Finally, BNY‐RPM seemingly made cloacal contact with the female, which left the bower and flew away immediately after. We cannot be sure that BNY‐RPM transferred sperm during cloacal contact, but it seems possible given that the duration of the copulation was comparable to other copulations recorded in normal circumstances. Immediately after these chaotic moments, the bower owner attacked and chased away the subordinate males from the display arena. BNY‐RPM was regularly seen at bower #50 prior to this observation and was filmed repeatedly at this bower for the rest of the breeding season (on 20% of days of monitoring)—including on the same day at 07:37, 08:58 and multiple times in the afternoon alone on the bower, as well as on the following day in the presence of the bower owner. In the following year (July–December 2019), BNY‐RPM was again a “regular” subordinate male at bower #50 and was filmed at this bower on 15% of the days of monitoring.

**FIGURE 1:**
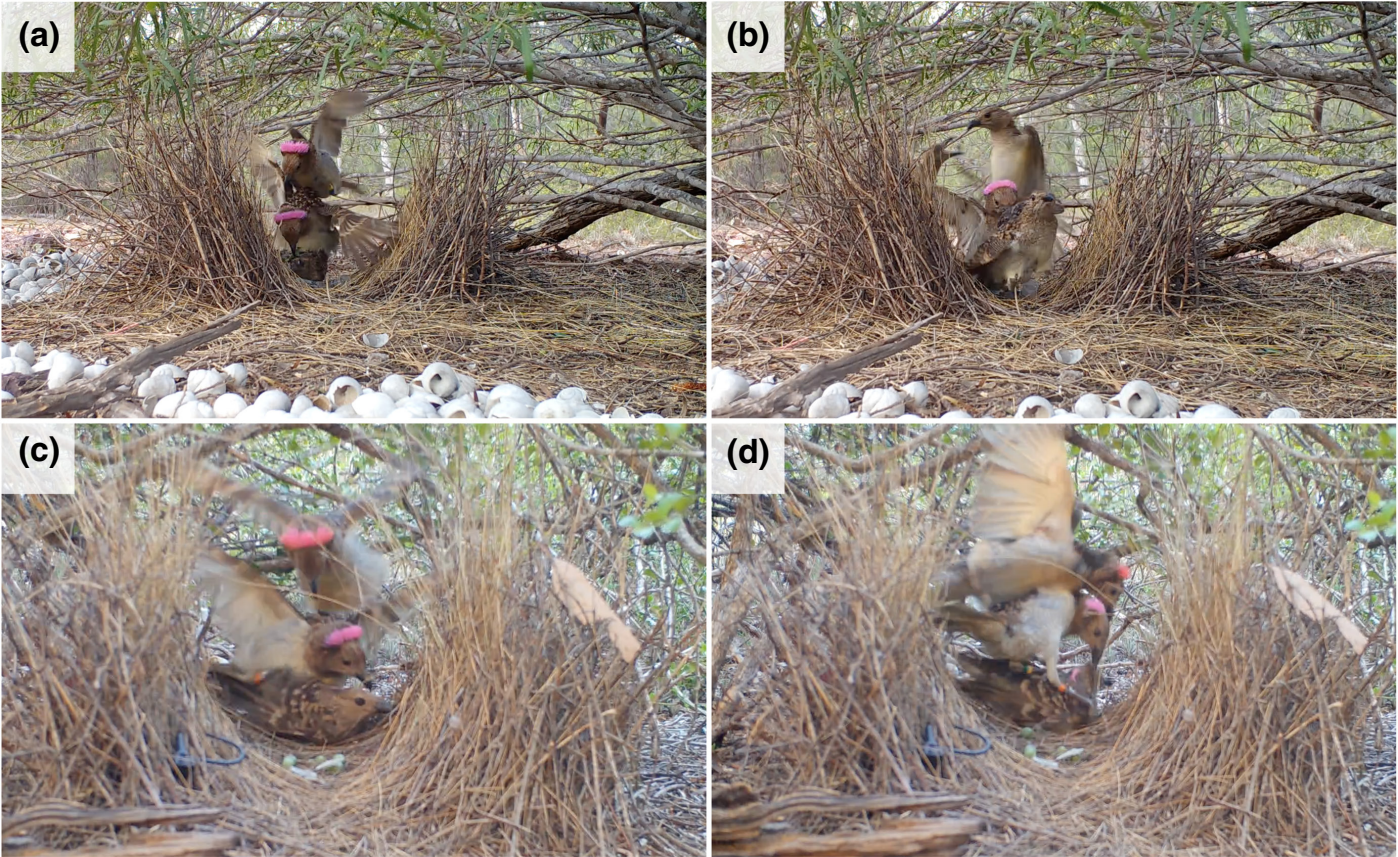
Screenshots of video recordings depicting copulation attempts by non‐resident males at bower #07 (a and b; Observation 2) and #23 (c and d; Observation 3). (a) A subordinate male identified with the colour bands BNY‐RPM flies inside the bower and interferes with the ongoing copulation between the resident male and an unbanded female. (b) Shortly after, a third (unbanded) non‐resident male joins BNY‐RPM and a fight ensues. (c) A non‐resident (unbanded) male attempts to copulate with a crouching female, immediately followed by the resident male. (d) The resident male violently attacks and wrestles with the non‐resident males on the display arena.

On 18 November 2018 at 07:49 (Observation 2), we video‐recorded at bower #7 (23.57833° S; 149.23831° E) the resident bower owner displaying on the arena, while an unbanded bird (presumably female) was crouching inside the bower as above. As soon as the bower owner mounted the crouching female (Figure 1a,b; Video S2), the subordinate male of bower #50 (BNY‐RPM, see above) and a third unbanded male flew on the bower and disrupted the ongoing copulation, attempting to mount the female themselves and fighting with the bower owner for a few seconds. In the confusion, BNY‐RPM seemingly made cloacal contact with the female, which left the bower and flew away immediately after. A violent aggression between the bower owner and the two other males ensued, with the former clawing at BNY‐RPM and plucking feathers. BNY‐RPM was not a “regular” subordinate male at bower #7; he was never recorded at bower #7 prior to this observation and was recorded again once after Observation 2 (3.8% of days of monitoring in total).

On 26 November 2018 at 05.33 (Observation 3), we video‐recorded at bower #23 (23.56965° S; 149.24881° E) an unbanded bird (presumably female) crouching inside the bower as above, while the resident bower owner was displaying. While the bower owner was off camera view (presumably displaying), an unbanded bird rapidly flew on the bower (Figure 1c,d; Video S3) and attempted a copulation. Immediately after, the bower owner interrupted the ongoing copulation, and attempted to mount the female, though the unbanded bird was interfering and attempting to displace the bower owner. Just after the female left the bower in the confusion that ensued from this fight, the resident male violently attacked the unbanded male, clawing at him and plucking feathers, until the unbanded bird flew off the field of view.

### 3.2 Observation of a copulation by an unbanded male individual in the absence of the bower owner

On 20 November 2018 at 08:38 (Observation 4) at bower #29 (23.54107° S; 149.23225° E), we video‐recorded an unbanded bird (presumably female) crouching inside the (partly destroyed) bower while the resident bower owner was displaying. At 8:39 am, after the bower owner left the camera field of view, an unbanded bird mounted the crouching female and copulated undisturbed (Video S4). The cloaca of the female was facing the camera after cloacal contact, and sperm was clearly visible on the cloaca, thus strongly indicating that sperm transfer did indeed occur. At 08:41 the bower owner was displaying again to an unbanded bird, but this courtship bout did not lead to a copulation.

### 3.3 Additional observations

We recorded two additional instances of copulation attempts in the presence of the bower owner, with similar dynamics as those described above. In both cases, however, the footage is fragmentary and the tarsi of the non‐resident individuals were not visible, thus preventing us from ascertaining their identity. On 15 November 2018 at 11:48 (Observation 5) at bower #7 (23.57833° S; 149.23831° E) the bower owner was displaying to a crouching female. At 11:49, a second individual (whose tarsi were not clearly visible) rapidly flew inside the bower while the bower owner was outside the camera field of view and attempted a copulation. The bower owner followed immediately after, and violently displaced the unknown bird from the female. We cannot be sure that any of the two birds succeeded in carrying out a full copulation, as one of the bower walls was covering the view. As in the observations above, a violent fight followed the departure of the female. On 25 November 2018 at 11:49 (Observation 6) at bower #11 (23.56499° S; 149.23518° E) we recorded a similar event involving the resident male and an individual with non‐visible tarsi, both of which attempted to mount a crouching female and fought on the display arena.

## 4 DISCUSSION

We reported here on four separate instances of sneaky copulations by non‐resident males in spotted bowerbirds during one breeding season. While the presence of subordinate males at established bowers has been documented in at least three bowerbird species (Madden, 2008), to the best of our knowledge no prior study on bowerbirds has documented potential reproductive output by males other than the bower owner. Extensive monitoring during previous research activities in spotted bowerbirds (J. R. Madden, personal communication) and great bowerbirds (L. A. Kelley, personal communication) did not record any similar event. Thus, much like in the majority of NRP birds, sneaky copulations by subordinate males appear to be very rare in bowerbirds. In manakins, there are also few reports of subordinate males with pre‐definitive (Araripe manakin *Antilophia bokermanni*, Gaiotti et al., 2020) or definitive plumage (long‐tailed manakin *Chiroxiphia linearis*, McDonald, 1989; white‐ruffed manakin *Corapipo altera*, Boyle & Shogren, 2019) gaining copulations and siring offspring (Rivers & DuVal, 2020; Schaedler et al., 2021). For instance, in white‐ruffed manakins (*Corapipo altera*), a single case of “cheating” by a beta male was reported in a recent paper (Boyle & Shogren, 2019).

The rarity of such events in spotted bowerbirds is remarkable, as extensive observations and monitoring have been conducted on this species by a number of researchers over several decades [>160,000 h of camera observation data on the same population between 2009 and 2011 by Isden (2014); see also Borgia (1995a, 1995b) and Madden (2002, 2003, 2006)]. Nonetheless, the fact that we recorded at least four independent observations involving different individuals strongly suggests that sneaky copulations were not an isolated and abnormal behaviour linked to exceptional ecological conditions, but plausibly a behavioural pattern or alternative reproductive strategy previously undocumented in subordinate males. Our observations, therefore, provide novel insights into male–male social dynamics in this species. However, as we were unable to document sneaky copulations on the following breeding season (July–December 2019) due to adverse environmental conditions, future studies should aim to replicate our observations in additional breeding seasons. Most importantly, it should be verified whether the observed sneaky copulations may be a conditional behaviour that is only expressed in certain (seemingly rare) conditions.

The fact that non‐resident males have been shown to occasionally increase their own reproductive success in a number of NRP species has important consequences for our understanding of same‐sex courtship coalitions. Indeed, tolerating other males on established display arenas may entail severe fitness consequences for resident males, even in the presence of short‐term benefits (Kokko & Johnstone, 1999). Thus, strict control over access to mates or retaliation against cheaters is predicted to evolve as a counter‐strategy, particularly in those systems where coalitions partners are unrelated to dominant males (Lebigre et al., 2014; Loiselle et al., 2007; McDonald & Potts, 1994). Indeed, our observations show that copulation attempts were always followed by violent aggressions on the display arena. Furthermore, past research showed an overall decrease of subordinate attendance across bowers during the breeding season, in particular after the onset of copulations by resident males (Isden, 2014; Spezie & Fusani, 2022). It seems, therefore, plausible that the rarity of sneaky copulations may be due to the stringent control exerted by bower owners on the arena and/or by the costs of retaliation. Nonetheless, Observation 1 involved a “regular” subordinate male with high attendance rates at that bower. Surprisingly, after this event we did not observe a change in the attendance rate of this subordinate male, and the owner of that bower continued to tolerate the presence of the subordinate male for the rest of the breeding season, as well as in the following one. One possible interpretation for the absence of retaliation is that the chaotic nature of sneaky copulations may have prevented the owner from identifying the individuals involved.

Some other aspects of our observations remain unclear. First, most observations involved unbanded individuals of unknown identity. Unbanded birds were indeed common across all bowers in both breeding seasons, due to the impossibility of catching and marking all individuals in the study population. Yet, unbanded individuals were on average less often observed at bowers than subordinate males (11.8 ± 6.7% of the total recording time per bower, versus 28.4 ± 13.5%, respectively; *N* = 14 bowers); therefore, it is surprising that a higher proportion of unbanded than banded subordinate males was involved in sneaky copulations. A possible alternative explanation is that sneaky copulations may be more frequently attempted by “floating” individuals that do not establish stable partnerships with resident males. This scenario raises the possibility that sneaky copulations may be a form of agonistic or disruptive behaviour by “floater” males. Indeed, non‐resident males would greatly benefit from attempting to usurp display sites from established bower owners. However, if that was the case, we would expect non‐resident males to exhibit agonistic behaviours towards resident bower owners and to interfere with the bower owners' reproduction in multiple other ways, for example by destroying their bower or fighting at the bower to try to usurp it. During two field seasons of data collection, we never observed non‐resident males destroying a bower, stealing decorations or challenging the bower owner by initiating a fight (unpublished data). Marauding behaviour in this species appears to only occur among resident males that own a display site (Madden et al., 2004).

We, therefore, speculate that males that do not own a display arena may pursue two alternative but possibly concurring strategies: (a) establishing stable partnerships with territorial bower owners to gain some delayed benefits (e.g. increased survival or bower inheritance, see above) or (b) act as “floaters” and attempt to gain sporadic access to females via sneaky copulations. While this hypothesis is intriguing, in at least one of our observations (Observation 1) copulations were attempted by a “regular” subordinate male of known identity; thus, these two strategies may not be mutually exclusive. In addition, subordinate males at an advanced developmental stage may attempt to usurp more copulations than younger coalition partners. Further data are required to investigate the hypothesis of alternative mating tactics in non‐resident spotted bowerbirds.

Finally, we cannot be sure that the observed copulations resulted in actual paternity. While during Observation 4 sperm transfer certainly occurred, it is well known that in a number of bird species females eject or select undesired sperm after mating (Birkhead & Møller, 1993; Birkhead & Montgomerie, 2020; Dean et al., 2011); therefore, sperm transfer does not provide conclusive evidence for paternity and direct fitness benefits. In particular, during the observations involving chaotic fights among males (Observation 1 to 3, 5, 6), it is plausible that females might have perceived bower visits as stressful events and may have not retained sperm and/or may have sought to obtain additional copulations. For instance, it has been shown that female fowl (*Gallus domesticus*) are more likely to eject sperm after sexual coercion by sub‐dominant males (Pizzari & Birkhead, 2000). Also, Rivers and DuVal (2020) showed that multiple mating in lance‐tailed manakins *Chiroxiphia lanceolata* is more common when females mate with beta or inexperienced males, but beta males do sire a small proportion of the offspring. Future studies should focus on female behaviour after sneaky copulations and investigate whether copulations by subordinate males result in multi‐male paternity in this species.

In conclusion, our observations rise interesting questions about male–male associations in bowerbirds and shed novel light on the costs that accrue to resident males from tolerating subordinate individuals at their bowers. We suggest that subordinate males are sexually mature individuals that obtain occasional access to females before establishing their own display arena (or inheriting it), and the observed sneaky copulations may represent an alternative reproductive strategy.

## AUTHOR CONTRIBUTIONS

Giovanni Spezie and Leonida Fusani developed the conceptual framework of the study. Giovanni Spezie conducted data collection and wrote the manuscript. Leonida Fusani reviewed and edited later versions of the manuscript.

## FUNDING INFORMATION

This study was funded by the Austrian Science Fund (FWF): W1262‐B29.

## CONFLICT OF INTEREST

The authors declare that they have no competing interests.

## Supporting information


Video S1

Video S2

Video S3

Video S4

## Data Availability

There are no data associated with this article.
